# Histoplasmosis of the Head and Neck Region Mimicking Malignancy: A Clinic-Pathological Predicament

**DOI:** 10.5146/tjpath.2022.01585

**Published:** 2023-05-15

**Authors:** Neha Mittal, Asawari Patil, Priyamvada Singhal, Munita Meenu Bal, Swapnil Ulhas Rane, Shivakumar Thiagarajan

**Affiliations:** Department of Pathology, Tata Memorial Center, Mumbai, India; Homi Bhabha National Institute, Mumbai, India; Department of Pathology, Subharti Medical College, Uttar Pradesh, India; Homi Bhabha National Institute, Mumbai, India; Department of Head and Neck Surgical Oncology, Tata Memorial Center, Mumbai, India

**Keywords:** Head and neck, Histoplasmosis, Granuloma, Histopathology, Pseudoepitheliomatous hyperplasia

## Abstract

***Objective:*** Histoplasmosis is a systemic, deep mycotic infection caused by Histoplasma capsulatum. Disseminated histoplasmosis (DH) is synonymous with HIV seropositive immunocompromised individuals; however, isolated histoplasmosis involving the head and neck mucosal sites mimicking malignancy is a clinical predicament. The result, in a superficial biopsy with marked pseudoepitheliomatous hyperplasia (PEH), in a tertiary care cancer center where the number of squamous carcinomas far outnumber the infectious diseases, could be catastrophic.

***Material and Method:*** The archives of a tertiary care cancer hospital were searched (2010-2019) for cases of histoplasmosis involving the head and neck mucosal sites in HIV non-reactive patients.

***Results:*** Six cases of isolated head and neck histoplasmosis were seen in biopsies from 4 men and 2 women, with an age range of 46-72 years. Three of these patients suffered from chronic illnesses. The most common site involved was the larynx (vocal cords) in three cases, two cases were involving lips, and one involving the tongue. The biopsies were reviewed in-house with a clinical diagnoses of malignancy in all and an outside biopsy diagnosis of “squamous cell carcinoma” in 2 cases. The important histological findings in the biopsy were PEH (3 cases), granulomas (2 cases), lymphoplasmacytic inflammation (all cases). Eosinophils were conspicuous by their absence. Intracellular histoplasma was seen in all cases, albeit to varying density, which was confirmed with GMS stain.

***Conclusion:*** A high index of suspicion, meticulous history taking by oncologists, and appropriate distinction of PEH from neoplastic squamous proliferation by pathologists in superficial biopsies and an apropos deeper wedge biopsy are essential to clinch the correct diagnosis.

## INTRODUCTION

Histoplasmosis, also known as “Darling’s disease” (1), “Cave’s disease” (2), and “Ohio valley disease” (3), is a systemic fungal infection caused by the thermally dimorphic fungus *Histoplasma capsulatum*. This fungus exists as hyphae in the soil environment at a temperature below 35°C and yeast forms in host tissue at 35-37°C (3), a typical climate of fertile river valleys. The usual route of transmission is through inhalation of spores in bird or bat droppings found in moist and acidic and nitrogen-rich soil (2). Construction-related or even recreational activities which involve disturbing the top 20 cm layer of the soil can lead to the release of the infectious conidia in the air, which can be inhaled (4). These yeast forms then replicate within the reticuloendothelial system and disseminate in the absence of a good immune status (4). Clinically, histoplasmosis can occur in three forms: [1] primary acute onset pulmonary form, [2] chronic pulmonary form, and [3] disseminated form (5,6). The disseminated form of histoplasmosis is practically synonymous with HIV infection and has been an AIDS-defining infection since 1987 (7). Histoplasmosis infection is often classified as being present in an HIV positive or HIV negative individual, as there are differences in presentation and response to therapy (6,7). However, isolated mucocutaneous or mucosal presentation in HIV non-reactive patients, which may clinically and histologically simulate malignancy, is rare and often less reported. The commonly involved sites in the head and neck are tongue, larynx, hard and soft palate, buccal mucosa, gingiva, and lips (8–11). Herein, we report a series of six cases of isolated histoplasmosis of head and neck mucosal sites which presented to a tertiary care cancer center in view of their clinical resemblance to malignancy. Notably, two of these had a histological diagnosis of malignancy from outside laboratories.

## MATERIALS and METHODS

The study has been conducted in compliance with the Declaration of Helsinki, and complies with the institutional medical ethical standards. The study was approved by the Institutional review board (project no. 900886).

We retrieved cases of histoplasmosis involving the head and neck region where histopathological material was available for evaluation from the archives of the surgical pathology department of a tertiary care referral cancer center. A total of 8 cases of head and neck histoplasmosis were retrieved over a nine-year period (2010-2018). Of these, in 2 cases, head and neck mucosal involvement was seen as a part of disseminated systemic histoplasmosis (DH) in HIV-positive patients; hence these cases were excluded. Only cases of isolated head and neck involvement in non-HIV reactive patients were included in this study. The relevant clinical details of patients were taken from hospital electronic medical records, including demographic details, history of co-morbidities including diabetes, other chronic illnesses, general and local clinical examination details. In all the cases, only biopsies were received for histopathological examination. The biopsies were reviewed by 2 head and neck pathologists for detailed histomorphological findings, including the site of biopsy, presence of pseudoepitheliomatous hyperplasia (PEH), type (lymphoplasmacytic, neutrophilic or eosinophil-rich), and degree of inflammation, type of tissue reaction (granulomatous or not), presence, location (intra- or extra-cellular) and a semi-quantitative estimation of the density of organisms. The density of fungal organisms was evaluated on H & E stained slide and labeled as ‘low’ when only scattered organisms were seen in a 40x field; and ‘high’ when an entire 40x field was full of intracellular histoplasma organisms.

## RESULTS

**Figure 1:**
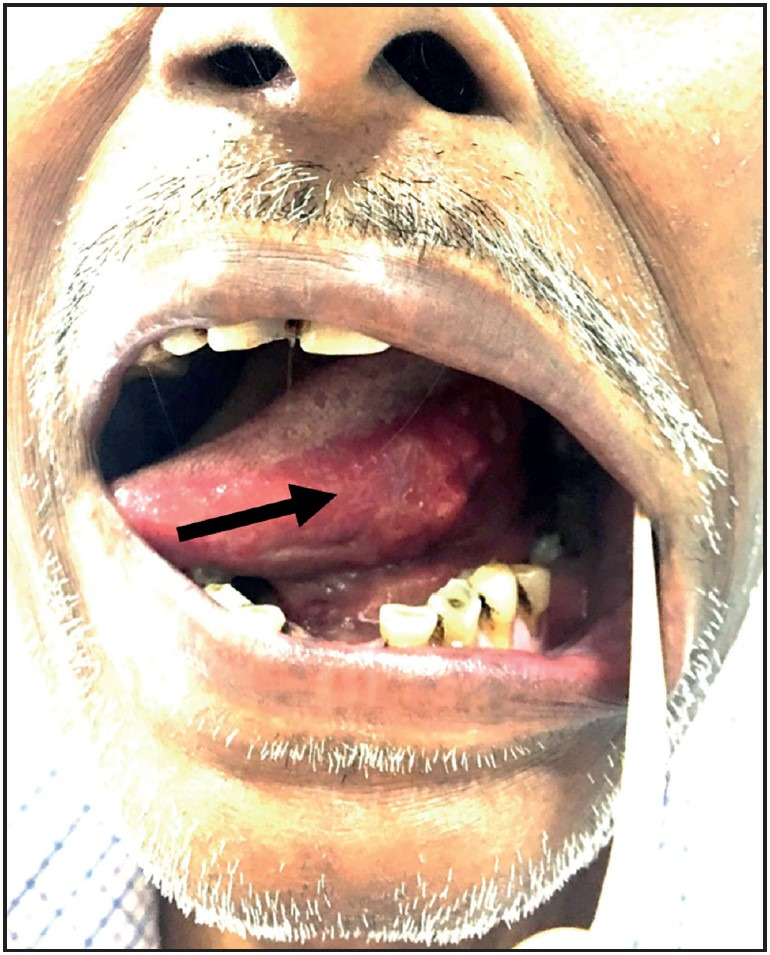
Clinical picture of Case 5. Ulcerated and indurated lesion on the left lateral border of tongue mimicking a malignant lesion on clinical examination.

A total of six cases of histoplasmosis involving head and neck region in HIV non-reactive individuals were found. Out of six, four were males and two females. The demographic features, site involved, associated medical illness, treatment, and outcome of these cases are described in Table 1. The median age of the patients was 59 years, with a range of 46-72 years. The most common site involved was the larynx (vocal cords) in three cases, along with two cases involving the lip and one involving the tongue (Figure 1). All the patients were seronegative for HIV; however, covert immunosuppression was found on detailed history taking in all three cases in which clinical information was available. Of these three patients, one had alcoholic liver disease (Case 3, Policeman by occupation), one patient suffered from chronic renal failure and was on dialysis (Case 5, Grocery shop owner), and one had uncontrolled diabetes mellitus, type II (Case 6, Homemaker). All these cases were clinically diagnosed as ‘cancer’. Also, two patients were diagnosed with “squamous cell carcinoma” on biopsies that were done and reported at an outside center.

**Table 1:** Demographic and clinical details of cases.

**Case No.**	**1**	**2**	**3**	**4**	**5**	**6**
Age/ sex	53 /M	46/M	60/M	70/F	58/M	72/F
Profession /occupation	Not Known	Not Known	Policeman	Not Known	Grocery shop owner	Homemaker
Symptoms	Hoarseness of voice	Hoarseness of voice	Hoarseness of voice and pain during swallowing	Mouth ulcer and swelling of lips	Ulcer on the tongue	Ulcer on left upper lip
Site involved	Left vocal cord	Both vocal cords	Bilateral arytenoids and vocal cords	Right oral commissure	Left side of tongue	Left upper lip involving angle of mouth
Clinical diagnosis	Carcinoma Glottis T1bN0M0	Carcinoma Glottis T1bN0M0	Carcinoma Larynx T2N0M0	Carcinoma right commissure T2N0M0	Carcinoma tongue	Diagnosed from outside as Sebaceous cell carcinoma
Associated medical illness	Nil	Incidentally noted bilateral pleural effusion	Alcoholic liver disease with Splenomegaly and moderate ascites	Nil	Chronic renal failure with Hypertension	Diabetes mellitus with Hypertension
Treatment	Antifungal	Antifungal	Antifungal	Antifungal	Antifungal	Antifungal
Follow up	Not available	Not available	Not available	Not available	Responded well, no relapse, Died of Chronic renal failure 18 months after diagnosis	Complete response, no relapse, died 10 months after diagnosis of Diabetes related complications.

**Figure 2:**
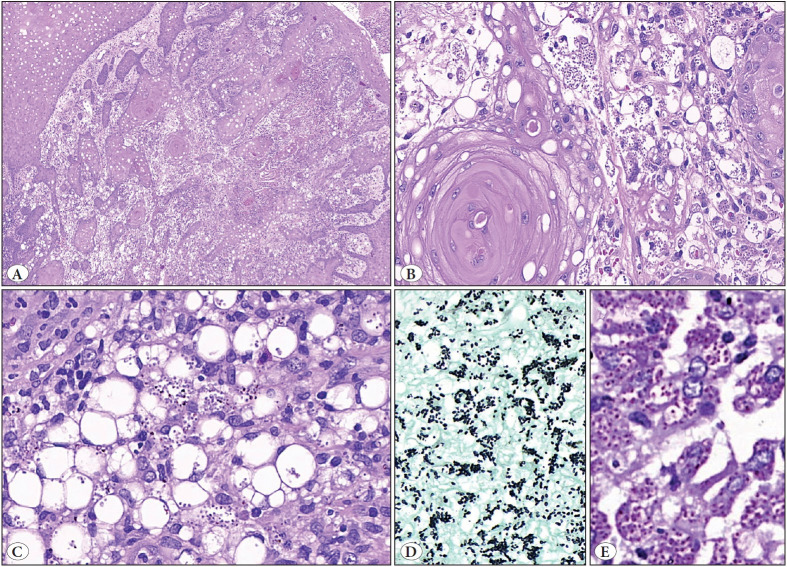
Histopathological findings of histoplasmosis (usual, non-granulomatous type). A case of oral histoplasmosis (Case 5) with prominent pseudoepitheliomatous hyperplasia. **A)** Prominent pseudoepitheliomatous hyperplasia of the squamous mucosa with small groups and tongues of squamous epithelium appear to infiltrate into the underlying submucosa, which closely resembles squamous cell carcinoma on low power (x100). **B,C)** Many foamy macrophages scattered in between the squamous islands **(B)** and in the submucosa **(C)**, which are teeming with intracellular organisms of 2-4 microns (x200). These organisms are strongly positive for GMS **(D)** and PAS **(E,** conforming to the morphology of Histoplasma).

**Figure 3:**
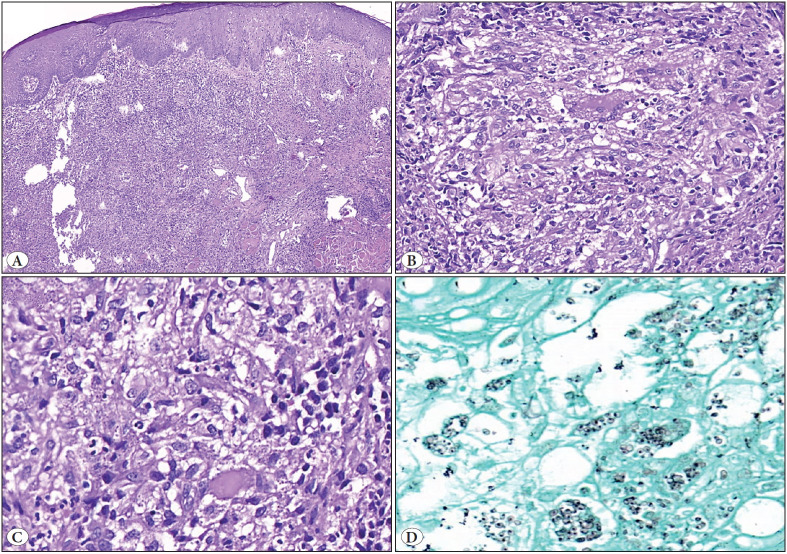
Histopathological findings of histoplasmosis with a granulomatous host response. A case of the less common type of histoplasmosis with a granulomatous host response (Case 2) and only a few yeast forms; a potential pitfall. **A)** Low power view to show relatively less prominent pseudoepitheliomatous hyperplasia with only mildly hyperplastic squamous mucosa(x100). **B)** Submucosal region showing an ill-formed epithelioid cell granuloma with giant cells, and epithelioid histiocytes (x200). **C)** Another area in the same biopsy with an admixture of epithelioid and foamy histiocytes; the foamy histiocytes show few intracellular organisms, which are highlighted by GMS **(D,** x200).

The histopathological features of these cases were given in Table 2 and Figure 2 and Figure 3. To summarize, in the biopsy, the overlying mucosa showed PEH in four cases (Case 1, 2, 5 and 6). Submucosal dense inflammation rich in histiocytes was seen in all cases, albeit to varying degrees. The histiocytic response seen was of two types; one type comprising foamy histiocytes distended with numerous yeast forms of intracellular fungal organisms (Case 3, 4, 5 and 6) and the second showing epithelioid to spindled histiocytes with very few intracellular microorganisms. Granulomas were seen in two cases. Eosinophils, which usually are a useful pointer towards fungal infection, were conspicuous by their absence. Gomori’s Methenamine Silver (GMS) and Periodic acid-Schiff (PAS) stains were done in all the cases and highlighted the morphology of *Histoplasma capsulatum* in the form of predominantly intracellular, 2-4 micron organisms with a pseudocapsule. Biopsy diagnosis was possible in all the cases. In two cases, biopsies were done outside and reported as malignancy (squamous cell carcinoma) and were sent to our center for a second opinion. The correct diagnosis was rendered upon review in our institute. Follow-up is available in only two cases (Case 5 and 6), and both of these patients responded to 4 weeks of antifungal therapy (Itraconazole) with complete resolution of the lesions. However, both these patients succumbed to their chronic underlying illness (Chronic renal failure and Diabetes mellitus) and died 18 months and 10 months post-treatment for histoplasmosis. The rest of the patients were referral cases.

**Table 2:** Histopathological features of Head and Neck histoplasmosis.

**Case No.**		**1**	**2**	**3**	**4**	**5**	**6**
**Overlying mucosa **		PEH	PEH	Ulceration	Ulceration	PEH with ulceration	PEH
**Submucosal region**							
Histiocytic cells	Epithelioid cells	In sheets	Ill formed granulomas	In sheets	Epithelioid granulomas.	In sheets	In sheets
	Foamy histiocytes	Few	Few	Many	Many	Many	Many
Fungal organism density on H&E*		High	Low	High	High	High	High
Giant cells		Nil	Occasional	Nil	Nil	Nil	Few
Other inflammatory cells		Few lymphocytes	Few plasma cells and lymphocytes	Plasma cells and few neutrophils	Plasma cells in sheets and few neutrophils	Plasma cells and lymphocytes	Lymphocytes and plasma cells

*Method of semi-quantitative estimation of fungal organism density is detailed in the “Materials and Methods section”

## DISCUSSION

Histoplasma capsulatum is a dimorphic fungus with damp soil (commonly near river beds) as its environmental reservoir. Pulmonary infection and disease as the most common presentation in humans. Though well-known now, these facts took decades to prove (10). This clinical disease was first described by Samuel Darling in 1905 while performing an autopsy on a patient in Panama (Central America) (1). The organism is unencapsulated; however, it produces a characteristic halo/clear zone in tissues, which was misinterpreted by Darling as a capsule and hence the name (1). Oral manifestation of histoplasmosis was first reported by Hansmann and Schenken in 1934 (7). In India, histoplasmosis is rare and is endemic only in small regions of West Bengal and Maharashtra (6,9). Panja and Sen described the first case of histoplasmosis involving the skin and viscera from Kolkata (12). Most of the patients have been reported from Northern or North-eastern states with endemic areas around the three main rivers, namely Brahmaputra, Ganga, and Yamun a (13). Cases from Western and Southern India have been in the form of few case reports and short series (11–14). However, an increasing number of cases of Disseminated Histoplasmosis are being reported from all over India in the last decade (14). Increasing awareness among physicians, marked urbanization with construction-related activities disturbing the layers of soil, and the increasing number of HIV-positive patients may all be deemed responsible (14). However, isolated mucosal involvement is still a rare and most plausibly under-reported occurrence.

Fewer than 5% of patients exposed to the infection contract an asymptomatic and self-limiting pulmonary disease known as “Asymptomatic Pulmonary Histoplasmosis”, following which the infection passes onto the reticuloendothelial system and skin (5). This is responsible for the disseminated forms of the disease, especially in HIV-positive patients (8). The involvement of the head and neck region may occur as a part of disseminated disease (15,16) (25-45% disseminated cases have oral cavity involvement) or rarely as isolated, sporadic involvement of the head and neck region which may or may not be associated with immunosuppression. In one of the reported series from Brazil, 9 out of 11 patients showed oral histoplasmosis as the first manifestation of disseminated histoplasmosis, with five having HIV seropositivity (17). However, occurrence in non-HIV infected hosts is worth reporting as fungal infections, in these patients, may be overlooked and misdiagnosed. The clinical resemblance of mucosal histoplasmosis and mucosal cancers adds to the conundrum, especially in dedicated oncology centers where the numbers of malignancies far exceed the cases with a benign /infectious etiology (15). Availability of clinical information regarding HIV seropositivity forewarns clinicians and pathologists to suspect unusual causes of infection. However, hidden chronic immunosuppressive conditions such as extremes of age, diabetes, alcoholism, and toxic therapies for malignancies may not be considered as harbingers of the same. Many of the patients do not have any identifiable risk factors for Histoplasmosis, particularly in a non-endemic area (15). Even though all our cases were seronegative for HIV, one case each had alcoholic liver disease (case 3), chronic renal failure (Case 5) or diabetes (Case 6), which are well-known causes of immunosuppression. In three cases, as they were only referral cases, history suggestive of immunosuppression could not be elucidated.

Clinically, mucosal histoplasmotic lesions are seen as firm, painful ulcers with verrucous, necrotic and polypoid proliferations which may be accompanied by regional lymphadenopathy, strongly simulating squamous cell carcinoma (17–20). This was true for all our cases as well, wherein all the cases presented with ulcerated lesions and were clinically suspected to be malignant lesions of their respective sites. Histologic findings of histoplasmosis are equally perplexing. Pseudoepitheliomatous hyperplasia (PEH) of the overlying mucosal epithelium is a common feature that may potentially mislead an unwary pathologist. The distinction of PEH from squamous cell carcinoma in a superficial biopsy with overwhelming inflammation, which is not uncommon in ulcerated mucosal squamous carcinomas, often presents diagnostic challenges to even the most experienced pathologists. Pseudoepitheliomatous hyperplasia is a benign condition characterized by hyperplasia of the epidermis seen in response to a wide variety of conditions, including infections, neoplasia, inflammation, and trauma, and it closely mimics squamous cell carcinoma (21). PEH of varying degrees was seen in 67% (4 out of 6 cases) of our cases and was particularly worrisome in 2 cases (cases 5 and 6). While the squamous epithelium of PEH can appear infiltrative and atypical; however, careful evaluation of histological features is usually sufficient to differentiate PEH from a squamous carcinoma. It is a known fact that atypical mitoses, lymphovascular invasion, and perineural invasion are never seen in PEH, and dyskeratosis is an exceedingly rare phenomenon in PEH. Two of these features, i.e., Atypical mitoses and dyskeratosis, are of value even on small biopsies (21–23). Nonetheless, exceptional difficulty may be encountered in differentiating the above two in small biopsies, and immunohistochemistry for p53, Matrix metalloproteinase 1, and E-cadherin have been found to be useful (24). In one of the case series from the same institute (2007), five cases of histoplasmosis were reported, which had clinically presented as carcinoma involving the oral cavity (3 cases), hypopharynx (1case), and larynx (1 case). Out of the five cases, three were mistaken for squamous cell carcinoma due to florid PEH of the overlying epithelium on a superficial biopsy. Two of these patients underwent radical treatment in the form of partial alveolectomy and hemiglossectomy with node dissection, respectively. This study concluded that a deep wedge biopsy is recommended to demonstrate the organisms in the subepithelial tissue, especially those with overt or hidden immunosuppression (18). The policy of deeper wedge biopsies was adopted since then, and all the cases in our study were diagnosed to have histoplasmosis on biopsies, and none of the patients underwent unnecessary radical surgery.

Goodwin et al. described three types of tissue reactions in histoplasmosis: diffuse histiocytosis, focal histiocytosis, and tuberculoid granulomas. Diffuse histiocytosis is one of the characteristics of disseminated histoplasmosis; focal histiocytosis occurs in moderate to severe degrees of infection, clinically presenting as oropharyngeal ulcers with an area of central necrosis. Tuberculoid granulomas are seen when the number of microorganisms in tissue macrophages is too small, indicating near normal tissue response and nearly normal immunocompetence (25). Four of our cases (cases 1, 3, 5 and 6) showed focal histiocytosis (Figure 2), and the rest of the two cases (cases 2 and 4) showed tuberculoid granulomas (Figure 3). One of these patients had the lowest density of fungal organisms amongst all. All our cases typically showed diffuse lymphohistiocytic infiltrate with fungal elements about 2-4 μm in size detected within the cytoplasm of histiocytes which were highlighted on GMS stain. A classic “halo” appearance caused by the cytoplasm retracting from the thick cell wall is helpful in identifying the fungi (4). Special stains like GMS stain and PAS stain highlight the fungi as the capsule of yeast is a polysaccharide and stains poorly with H and E stain (5,6). All our cases showed intracellular fungi on GMS stain.

To summarize, if suspected, the diagnosis of head and neck histoplasmosis is achievable with the basic tools available in many histopathology laboratories. Thus, it is advisable that pathologists should refrain from over-diagnosis of malignancy on small-sized biopsies, superficial biopsies with improper orientation, and dense inflammatory changes. Such situations necessitate the request for deeper sections or repeat deeper biopsies. Fulfillment of stringent malignant features should always remain the cornerstone for the diagnosis of cancer.

## CONCLUSION

The cases in this series hold a very important “back to basics” lesson for the clinicians as well as the pathologists. The importance of a meticulous history and thorough physical examination, even for obvious localized diseases, should not be underscored by clinicians, including oncologists. The fundamentals of neoplasia and its distinction from inflammation-related atypia on histology are indelible lessons for pathologists to prevent any unnecessary treatment to the patients.

## Conflict of Interest

The authors declare no conflict of interest.
